# Treatment patterns in stage III non‑small‑cell lung cancer patients: a population‑based study using German cancer registry data

**DOI:** 10.1007/s00432-023-05289-7

**Published:** 2023-08-30

**Authors:** Ahmed Bedir, Sneha Mehrotra, Jessica Gnüchtel, Dirk Vordermark, Daniel Medenwald

**Affiliations:** 1 Department of Radiation Oncology, Health Services Research Group, University Hospital Halle (Saale), Ernst-Grube-Str. 40, 06120 Halle (Saale), Germany; 2 Faculty of Life Sciences and Medicine, King’s College London, Guy’s Campus, London, SE1 1UL UK; 3 Department of Radiation Oncology, University Hospital Halle (Saale), Ernst-Grube-Str. 40, 06120 Halle (Saale), Germany; 4 Department of Traumatology, Elisabeth-Hospital Leipzig, Biedermannstraße 84, 04277 Leipzig, Germany

**Keywords:** Lung cancer, Treatment patterns, Germany, Cancer registries, Survival analysis

## Abstract

**Purpose:**

Lung cancer remains the leading cause of cancer-related mortality worldwide, mostly due to delayed diagnosis. The objective of this study is to examine the treatment patterns and overall survival (OS) outcomes in a cohort of patients diagnosed with stage III non-small cell lung cancer (NSCLC) over a period of 12 years in Germany.

**Methods:**

This retrospective study is based on German cancer registry data and included 14,606 stage III NSCLC patients diagnosed during 2007–2018. Three time-periods were defined according to the availability of advanced diagnostic and treatment strategies (2007–2010 low availability era (LAE), 2011–2014 transition era (TE), 2015–2018 modern era (ME)). Patients were categorized according to the treatment they received during those eras. Kaplan–Meier curves and multivariate Cox proportional hazards models were used to investigate the association between being diagnosed during a certain era and survival. The hazard ratio (HR) estimates were reported along with the 95% confidence interval (CI).

**Results:**

The median OS rose from 16 months in the LAE to 22 months in the ME. The HR for patients diagnosed and treated in the ME was estimated to be [0.78; 95% CI (0.74–0.83)] compared to those diagnosed and treated in LAE. The benefit was most evident for patients treated by radiotherapy and chemotherapy [HR 0.73 95% CI (0.66–0.82)].

**Conclusion:**

This study highlights the importance of diagnostic and treatment advances in improving outcomes for lung cancer patients. Further studies are needed to assess progress in survival rates with current immunotherapy integration.

**Supplementary Information:**

The online version contains supplementary material available at 10.1007/s00432-023-05289-7.

## Introduction

Lung cancer remains the leading cause of cancer-related mortality worldwide, particularly due to the late diagnosis of patients at an advanced stage (III/IV) (Watanabe et al. 2017). Non-small cell lung cancer (NSCLC), which accounts for 85% of all lung cancer cases, is divided into four stages based on the TNM system introduced by the American Joint Committee on Cancer (Amin et al. 2017; Zappa and Mousa 2016). Stage III NSCLC describes a heterogeneous population of patients experiencing a ‘locally advanced’ stage of lung cancer that involves adverse prognostic features associated with the primary tumor and/or the existence of metastases within regional lymph nodes (Evison 2020; Pöttgen et al. 2017). According to the Robert Koch Institute, the 5-years relative survival of stage III NSCLC cases was estimated to be 27% and 20% in women and men respectively, compared to 7% and 4% for stage IV patients (Erdmann et al. 2021).

Optimal treatment for stage III NSCLC patients remains a topic of debate. While the current guidelines recommend concurrent chemoradiotherapy (CRT) as the ‘standard of care’ for the majority of stage III patients, factors such as, tumor sub-stage and resectability, along with the patient profile (lung function, comorbidities, and age), may all affect the treatment strategy and subsequently survival (Ettinger et al. 2022; Leitlinienprogramm Onkologie (Deutsche Krebsgesellschaft and AWMF) 2022). In order to determine the ideal treatment plan for a NSCLC patient, it is therefore imperative for the tumor to be accurately assessed.

The landscape of advanced lung cancer management has undergone significant transformation over the past decade, primarily due to advancements in diagnostic and treatment strategies. Key improvements include the use of F-fluorodeoxyglucose positron emission tomography (F-FDG PET/CT) imaging and intensity-modulated radiation therapy (IMRT), alongside changes in chemotherapy regimens, the introduction of immunotherapy, and the emergence of targeted therapies for specific genetic mutations (Brahmer et al. 2018; Hallqvist et al. 2017; Hu et al. 2016; Mäurer et al. 2022; Mok et al. 2019; Nestle et al. 2020; Planchard et al. 2022; Sura et al. 2008). These advancements have led to measurable improvements in survival outcomes for patients diagnosed with non-small cell lung cancer (NSCLC).

Considering these developments, the aim of this study was to (1) describe the patterns of treatment in a cohort of patients with stage III NSCLC over different time periods and (2) investigate if being diagnosed and treated in recent time periods resulted in an improvement in overall survival.

## Materials and methods

### Data source and study population

This retrospective study is based on German population-based cancer registry data, collected and sent annually to the German Centre for Cancer Registry Data (Zentrum für Krebsregisterdaten, ZfKD) at the Robert Koch Institute (RKI) (Kraywinkel et al. 2014). Upon receiving the data, the ZfKD then undergoes data quality checks before producing a pooled anonymized dataset available for research purposes. The pooled dataset contains information on gender, month and year of birth, date of diagnosis, tumor topography and morphology, tumor grading and stage, and data on delivered treatments and death events.

This study used pooled nationwide NSCLC data, representing all patients diagnosed in Germany from 2007 to 2018. State cancer registries were included only if the overall proportion of Death Certificate Only (DCO) cases did not exceed 13% in 2007–2018 (EUROCARE-5 study) (Rossi et al. 2015). In addition, cancer registries that did not record treatment data or had a high percentage of its treatment information missing were excluded. Only six federal states (Baden-Württemberg, Saarland, Brandenburg, Mecklenburg–Western Pomerania, Saxony, and Thuringia) met the inclusion/exclusion criteria.

Patients diagnosed with stage III NSCLC (International Classification of Diseases for Oncology-ICD C34) during the period of 2007–2018 were included in our analysis. Stage III was defined using the Union for International Cancer Control TNM Classification 8th edition and was further categorized into IIIA, IIIB and IIIC (Amin et al. 2017). The different histological subtypes of NSCLC were identified through their respective ICD-0 histology codes (Bray et al. 2021).

Cases were excluded if they were diagnosed by means of an autopsy, DCO, or had missing information on survival status and exact date of death. In addition, patients with a prior history of lung cancer or multifocal lung cancers were also excluded.

In our dataset, information on treatment was available as a binary variable (surgery yes/no, radiotherapy yes/no, chemotherapy yes/no). We categorized treatment into six groups: Surgery only, Surgery + Radiotherapy, Surgery + Chemotherapy, Radiotherapy only, Radiotherapy + Chemotherapy, Chemotherapy only, and Surgery + Radiotherapy + Chemotherapy. Since our study is focused on treatment patterns, untreated cases or cases with missing treatment information were not included in our analysis.

### Definition of time-periods

The time-periods in our study are defined based on key developments in NSCLC diagnosis and treatment in Germany. The low availability era (2007–2010) corresponds to the initial implementation of FDG-PET for treatment planning (Bundesausschuss 2007). The ‘transition era’ (2011–2014) represents the period of growing utilization of IMRT. By 2011, IMRT was employed in over 10% of treated lung cancer cases, a substantial increase from less than 5% in 2008, and its usage continued to escalate, exceeding 25% by 2013 (Forschungsdatenzentrum 2016). Finally, the modern era (2015–2018) represents a period when both FDG-PET and IMRT were widely available and utilized, signifying the most advanced diagnostic and treatment methods available during the study period.

### Outcome measure

Our primary outcome was overall survival (OS), measured from the date of cancer diagnosis to the date of death from any cause. Patients lost to follow-up before death or still alive at the last vital status assessment were right-censored at the date of the last vital status assessment or at the censor date (31 December 2018).

### Statistical analysis

Demographic and clinical characteristics according to the time-period groups were described using common descriptive statistics. Differences between these groups were evaluated using appropriate statistical tests, with the resulting *p* values indicating the level of significance. The median OS and the observable 3-years overall survival rates were reported to describe the survival of patients diagnosed within a certain time-period and for patients in our predefined treatment groups. Kaplan–Meier curves were used to visualize and estimate the 3-years survival rates. Differences in survival across the time-period groups were assessed using the log-rank test. After stratifying patients according to their respective treatment group, we conducted a multivariate analysis using the Cox proportional hazards model to investigate the association between being diagnosed during a certain era and survival. The Cox model adjusted for sex, age, stage subtype, and histology. We also adjusted for the treatment variable in an additional model to measure the general effect of the time-periods on survival. The hazard ratio (HR) estimates were reported along with their respective 95% confidence interval (CI).

### Subgroup and sensitivity analysis

We conducted a subgroup analysis, stratifying our sample according to the sub-stages IIIA, IIIB, and IIIC as defined by TNM 8th edition. This updated classification, introduced in 2016, included the creation of sub-stage IIIC and revisions to the definitions of the existing sub-stages IIIA and IIIB. These changes could potentially lead patients diagnosed in the modern era with these two sub-stages, to appear to have better survival. To address potential bias, we conducted a sensitivity analysis, reapplying our multivariate analysis using the definitions of sub-stages from the 7th edition (Edge and Compton 2010). Furthermore, to address potential biases associated with the varied process of recording treatment information across the included cancer registries, an additional sensitivity analysis was performed, taking into account the different cancer registries included from both East and West Germany.

All analyses were conducted in R statistical software version 3.2.3 (Team 2013).

## Results

### Patients characteristics

In total, our analysis included 14,606 NSCLC stage III patients diagnosed between 2007 and 2018 (Table 1). The mean age at diagnosis for the study population was 67.3 ± 9.9 years. The mean age was more or less the same across the three time-periods with the age distribution showing slight differences. While the majority of the patients were men (74%), the later eras showed an increase in the proportion of women patients reaching 28% in 2015–2018. The most common pathologies across the three time-periods were squamous cell carcinoma and adenocarcinoma. With regard to sub-stage present at diagnosis, 44.7% of the patients were diagnosed at IIIA, in comparison to 39.0% IIIB and 16.3% IIIC. The distribution of the sub-stages also showed no differences across the time eras.

**Table 1:** Characteristics of NSCLC stage III treated patients 2007–2018

	Total	2007–2010	2011–2014	2015–2018	*P* value
Number	14,606	3980	5493	5133	
Age mean (SD)	67.3 (9.9)	67.0 (9.9)	67.4 (10.0)	67.4 (9.9)	0.170
< 50	661 (4.5)	224 (5.6)	247 (4.5)	190 (3.7)	< 0.001
50–64	5134 (35.1)	1217 (30.6)	1969 (35.8)	1948 (38.0)	
65–79	7456 (51.0)	2235 (56.2)	2726 (49.6)	2495 (48.6)	
> 80	1355 ( 9.3)	304 (7.6)	551 (10.0)	500 ( 9.7)	
Gender *n* (%)					< 0.001
Women	3776 (25.9)	896 (22.5)	1432 (26.1)	1448 (28.2)	
Men	10,830 (74.1)	3084 (77.5)	4061 (73.9)	3685 (71.8)	
Clinical stage at diagnosis *n *(%)					0.365
IIIA	6524 (44.7)	1779 (44.7)	2452 (44.6)	2293 (44.7)	
IIIB	5696 (39.0)	1524 (38.3)	2132 (38.8)	2040 (39.7)	
IIIC	2386 (16.3)	677 (17.0)	909 (16.5)	800 (15.6)	
Histology *n* (%)					< 0.001
Squamous-cell carcinoma	7111 (48.7)	2000 (50.3)	2597 (47.3)	2514 (49.0)	
Adenocarcinoma	5440 (37.2)	1344 (33.8)	2052 (37.4)	2044 (39.8)	
Large cell carcinoma	453 (3.1)	182 (4.6)	167 (3.0)	104 (2.0)	
Other specified	1374 (9.4)	381 (9.6)	559 (10.2)	434 (8.5)	
Other unspecified	228 (1.6)	73 (1.8)	118 (2.1)	37 (0.7)	
Survival					
Median OS, months (95% CI)	18 (17–19)	16 (15–17)	17 (16–18)	22 (21–23)	
3-year survival, % (95% CI)	30.5 (29.7–31.3)	26.2 (24.9–27.6)	29.8 (28.6–31.0)	37.1 (35.4–39.0)	

### Treatment patterns

**Fig. 1:**
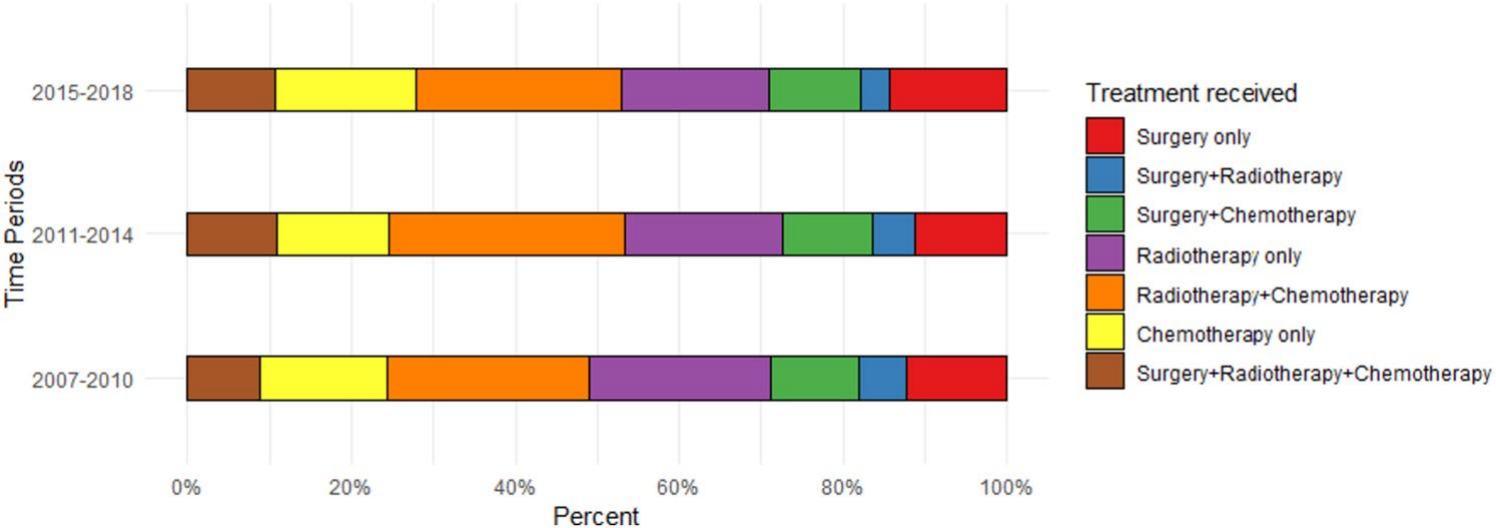
Bar plot showing distribution of treatment strategies of stage III NSCLC across the time periods

The three most common treatment modalities received by the patients across the three eras were: radiotherapy + chemotherapy, radiotherapy only, and chemotherapy only (Fig. 1). The bimodal treatment of surgery + radiotherapy was the least delivered mode of treatment. The administration of radiotherapy + chemotherapy, the tri-modal treatment of surgery + chemotherapy + radiotherapy, and treatment with “surgery only” and “chemotherapy only” increased over time. Surgery + chemotherapy + radiotherapy increased from 8.9% in 2007–2010 to 10.8% in the modern era, while surgery only increased from 12.3% to 14.4% respectively. Surgery + radiotherapy and radiotherapy only both decreased over time (Appendix 1).

### Survival analysis

**Fig. 2:**
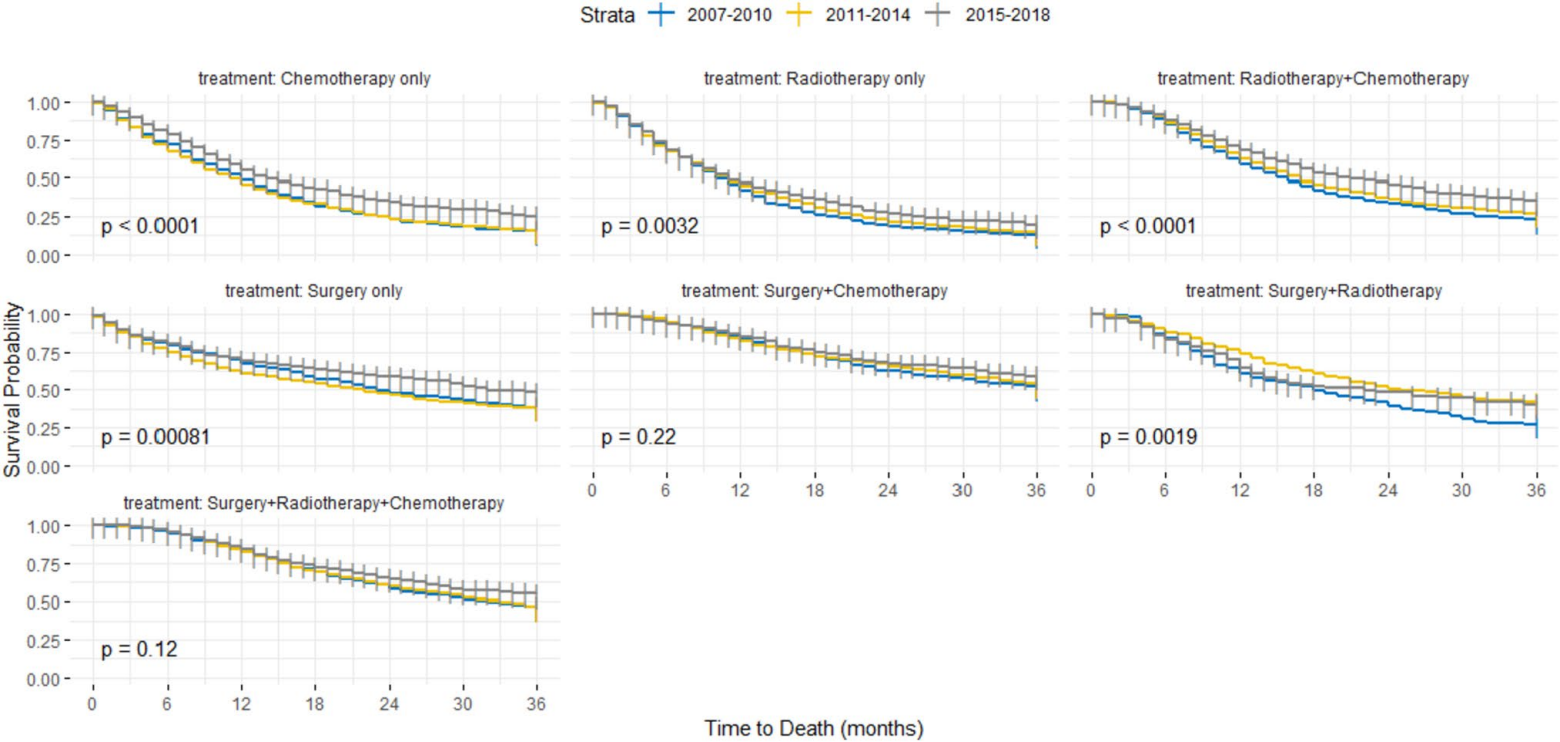
Survival according to treatment received across the three time-periods

Our entire sample showed a median OS of 18 months (95% CI 17.0–19.0). The observed 3-years OS was 30.5% (95% CI 29.7–31.3) (Table 1). The 3-years survival rate increased by more than 10 percentage points from the 2007–2010 time-period 26.2% (95% CI 24.9–27.6) to the modern era 37.1% (95% CI 35.4–39.0). This was also reflected in the median OS, which rose from 16 months (95% CI 15.0–17.0) in the low-availability era to 17 months (95% CI 16.0–18.0) in the transition era, to finally 22 months (95% CI 21.0–23.0) in the most recent era. Stratifying the cases according to treatment received across the time-periods, median OS estimates revealed the same pattern (Appendix 1). Patients diagnosed in the modern era always showed a better OS no matter which treatment they received (Fig. 2, Appendix 1). KM estimates demonstrated that patients who received chemotherapy only, radiotherapy + chemotherapy, and surgery + radiotherapy experienced the most significant improvement in survival (Appendix 1). Adjusting for age, sex, histology, and cancer sub-stage in our cox proportional-hazards model, the effect of time was clear (Table 2). All patients diagnosed and treated in the modern era consistently showed a lower hazard of dying when compared to our reference group (2007–2010) [HR 0.78 95% CI (0.74–0.83)]. This effect was strongest for patients treated by radiotherapy + chemotherapy [HR 0.73 95% CI (0.66–0.82)]. Further adjusting for the different cancer registries included did not alter our results (Appendix 2).

**Table 2:** Hazard ratios comparing patients diagnosed in 2011–2014 and 2015–2018 to reference group (2007–2010) stratified according to treatment received

	Surgery only	Surgery + radiotherapy	Surgery + chemotherapy	Radiotherapy only	Radiotherapy + chemotherapy	Chemotherapy only	Surgery + radiotherapy + chemotherapy	Total^a^
2007–2010								
2011–2014	1.06 (0.91–1.23)	0.66 (0.53–0.82)^*^	0.96 (0.80–1.15)	0.94 (0.86–1.04)	0.91 (0.83–0.99)^*^	1.02 (0.91–1.14)	0.97 (0.81–1.16)	0.94 (0.89–0.99)^*^
2015–2018	0.79 (0.67–0.92)^*^	0.74 (0.56–0.97)^*^	0.84 (0.69–1.04)	0.84 (0.76–0.94)^*^	0.73 (0.66–0.82)^*^	0.77 (0.68–0.88)^*^	0.82 (0.66–1.00)	0.78 (0.74–0.83)^*^

Cox models adjusted for age, sex, histology, and cancer sub-stage

^a^Adjusted for treatment in addition to baseline variables

^*^Indicates statistical significance

### Subgroup analysis

**Fig. 3:**
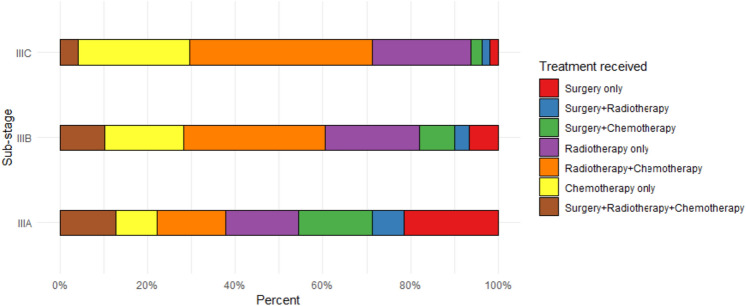
Bar plot showing distribution of treatment strategies of stage III NSCLC according to sub-stage during 2007–2018

**Fig. 4:**
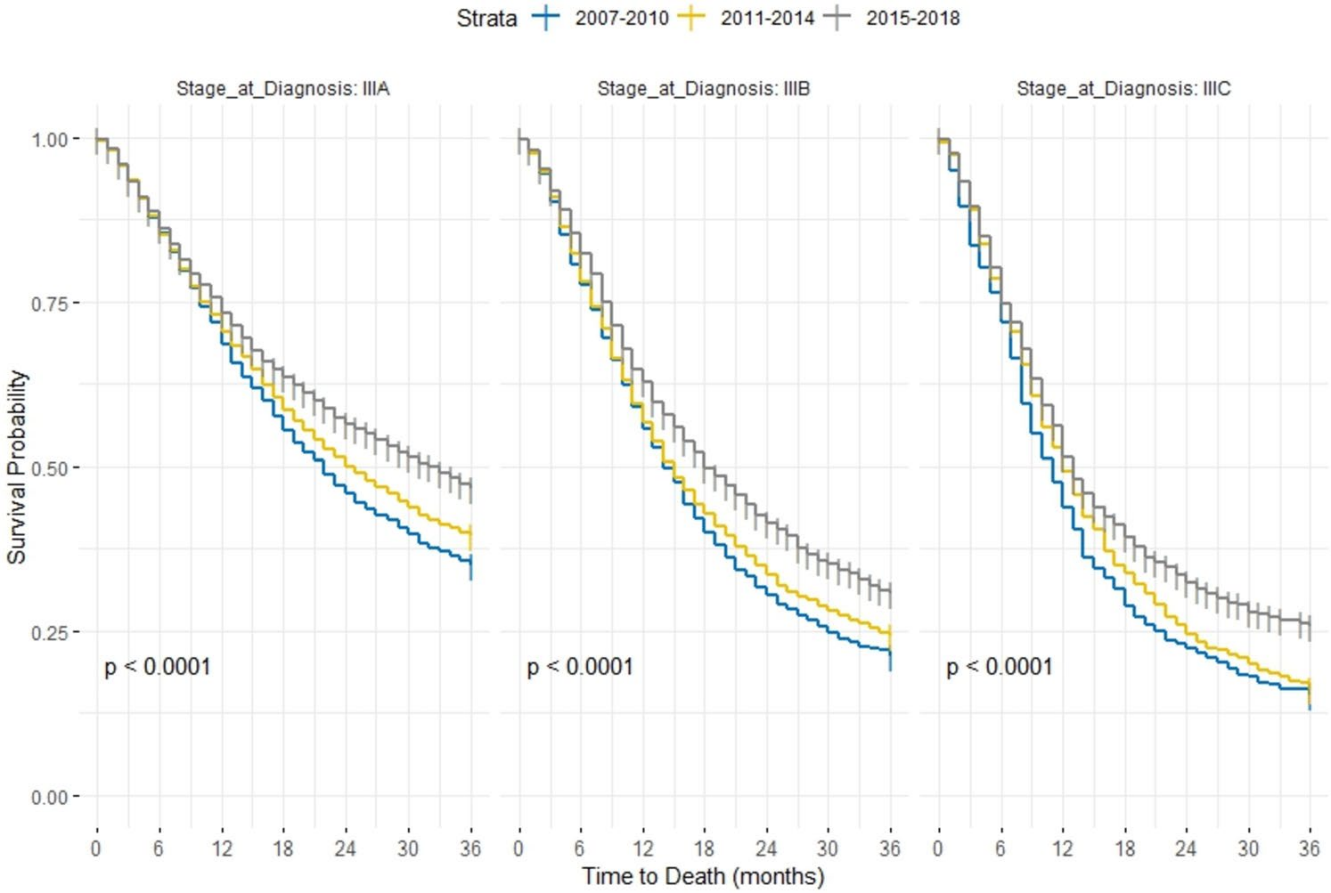
Survival according to cancer sub-stage across the three time-period

To address the different treatment strategies that are based on cancer sub-stage, we further stratified our sample according to the sub-stage present at diagnosis. Surgery only was the most common treatment that patients diagnosed with sub-stage IIIA received. Patients diagnosed at sub-stages IIIB and IIIC were more likely to receive radiotherapy + chemotherapy and chemotherapy only (Fig. 3). Similar to our unstratified estimates, the median OS and 3-year survival rates were always higher in patients diagnosed in the later eras, regardless of the sub-stage (Fig. 4, Appendix 1). Our cox proportional hazard models further highlighted this improvement in survival. Patients diagnosed in the transition and modern era consistently showed a lower hazard of overall mortality when compared to patients diagnosed in the early era, especially for patients receiving radiotherapy + chemotherapy at sub-stages IIIA [Modern era: IIIA HR 0.60, 95% CI (0.49–0.75)] and IIIC [Modern era: IIIC HR 0.69, 95% CI (0.56–0.85)] (Table 3). Changing how we defined the sub-stages using the 7th edition, did not alter our results (Appendix 2).

**Table 3:** Sub-stage stratified hazard ratios comparing patients diagnosed in 2011–2014 and 2015–2018 to reference group (2007–2010) stratified according to treatment received

	Surgery only	Surgery + radiotherapy	Surgery + chemotherapy	Radiotherapy only	Radiotherapy + chemotherapy	Chemotherapy only	Surgery + radiotherapy + chemotherapy	Total^a^
IIIA								
2007–2010								
2011–2014	1.05 (0.85–1.22)	0.65 (0.49–0.85)^*^	0.97 (0.77–1.22)	0.92 (0.79–1.09)	0.73 (0.61–0.88)^*^	1.11 (0.88–1.40)	0.96 (0.74–1.23)	0.91 (0.84–0.98)^*^
2015–2018	0.75 (0.61–0.90)*	0.66 (0.46–0.95)^*^	0.73 (0.55–0.96)^*^	0.93 (0.77–1.11)	0.60 (0.49–0.75)^*^	0.86 (0.67–1.10)	0.84 (0.62–1.12)	0.77 (0.70–0.84)^*^
IIIB								
2007–2010								
2011–2014	1.27 (0.92–1.73)	0.66 (0.45–0.98)^*^	0.99 (0.72–1.36)	0.93 (0.80–1.07)	0.98 (0.85–1.12)	1.00 (0.84–1.19)	0.86 (0.65–1.13)	0.96 (0.89–1.04)
2015–2018	0.95 (0.67–1.32)	0.89 (0.54–1.45)	1.10 (0.78–1.54)	0.78 (0.66–0.91)^*^	0.82 (0.70–0.96)^*^	0.71 (0.59–0.84)^*^	0.75 (0.55–1.03)	0.79 (0.73–0.86)^*^
IIIC								
2007–2010								
2011–2014	0.91 (0.37–2.24)	0.74 (0.39–1.91)	0.74 (0.33–1.67)	0.97 (0.78–1.20)	0.94 (0.79–1.12)	0.94 (0.76–1.17)	2.08 (1.01–4.25)	0.97 (0.87–1.08)
2015–2018	0.88 (0.39–1.98)	0.84 (0.35–1.99)	0.89 (0.35–2.20)	0.84 (0.66–1.07)	0.69 (0.56–0.85)^*^	0.82 (0.64–1.04)	1.27 (0.54–2.94)	0.79 (0.69–0.89)^*^

Cox regression models were adjusted for age, sex, and histology

^a^Adjusted for treatment in addition to baseline variables

*Indicates statistical significance

## Discussion

Our study describes the treatment patterns for stage III NSCLC patients in Germany between 2007 and 2018, while focusing on comparing the treatment modalities and overall survival outcomes of cases diagnosed and treated during different time-periods. Our findings showed an improvement in overall survival for patients diagnosed and treated in more recent years compared to those diagnosed earlier, with the largest improvement seen in patients treated with surgery + radiotherapy and radiotherapy + chemotherapy.

Considering that our study used time-periods as a proxy for improvements in diagnosis and treatment and our analysis only included treated cases, our results suggest that advancements in diagnostic tools like FDG-PET and treatment methods such as IMRT could have contributed to the observed improvements in survival. We base this assumption on findings from multiple studies that highlight the potential of these techniques in enhancing oncological outcomes and reducing treatment-related toxicity. For instance, a recent cohort study by the young DEGRO Trial Group assessed the effect of radiotherapy treatment planning on overall survival and found that in stage III NSCLC patients diagnosed during 2010–2013, the use of PET/CT resulted in better oncological outcomes (HR = 0.80, CI 0.56–1.16) (Mäurer et al. 2022). This was also presented in the findings of Nestle et al., where the authors found that F-FDG PET-based reduction of radiotherapy target volume decreased the risk of locoregional progression compared to conventional target (CT)-based planning [HR 0.57, 95% CI 0.30–1.06] (Nestle et al. 2020). Similarly, the growing application of IMRT in concurrent chemoradiotherapy has shown potential in enhancing survival by minimizing the risk of life-threatening toxicity through precise and targeted delivery, as pointed out by Sampath et al. (Sampath 2016).

The randomized phase 3 trial (RTOG 0617) trial has also revealed that standard-dose radiotherapy outperformed high-dose radiotherapy in terms of overall survival, possibly due to fewer treatment-related deaths or severe adverse events (Bradley et al. 2020; Bradley et al. 2015). Furthermore, a secondary analysis associated IMRT with lower rates of severe pneumonitis and cardiac doses, contributing to survival advantages due to reduced toxicity and treatment interruptions (Chun et al. 2017; Koshy et al. 2017). While we suggest that these advancements could explain the improved survival rates in the modern era, especially among the radiotherapy + chemotherapy group, it is also crucial to recognize other possible factors, like developments in chemotherapy regimens or the introduction of immunotherapy. Immunotherapy, recommended for NSCLC treatment in 2018 (Brahmer et al. 2018; Gubens and Davies 2019; Novello et al. 2023) could have contributed significantly to positive outcomes. However, our dataset lacks information on the specific type of chemotherapy administered to the patients and whether they received immunotherapy. This limitation prevents us from conducting a comprehensive investigation into their respective effects on survival rates.

To our knowledge, this is the first study to explore treatment patterns of stage III NCSLC in Germany. As cancer treatments are continuously progressing, the literature and guidelines are evolving, therefore the results from studies across Europe and lung cancer stages are often extrapolated from due to the use of different time-periods in our study. The 2022 update to the German S3 guideline for lung cancer advises that a comprehensive thoracic-oncological tumor board be established to determine the extent of additional diagnostics required, stage the cancer accurately, and make informed decisions on the best treatment for each individual patient (Leitlinienprogramm Onkologie (Deutsche Krebsgesellschaft and AWMF) 2022). The guidelines further recommend concurrent chemoradiotherapy (CRT) as the preferred treatment for most patients with stage III NSCLC, including those with inoperable tumors. Additionally, the combination of immunotherapy with CRT is advised for selected patients with a PD-L1 (Programmed cell death receptor ligand 1) expression level of 1% or more. Considering that our study sample was diagnosed over a 12 year time period (2007–2018), the trend towards the bimodal treatment modality of radiotherapy + chemotherapy as the preferred treatment for stage III NSCLC was evident. This group of patients, along with those treated with surgery + radiotherapy demonstrated the largest improvement in survival.

Our study also revealed considerable variation in treatment modalities across the different sub-stages of NSCLC. Most patients diagnosed at stage IIIA were treated with either surgery or a combination of surgery and chemotherapy. In contrast, about 75% of cases at stages IIIB and IIIC were treated with either radiotherapy + chemotherapy or radiotherapy alone. These findings were also in line with guideline-recommended treatment. Importantly, all sub-stages experienced an improvement in overall survival, indicating that advancements in diagnostic and therapeutic strategies are benefiting a wide range of patients. It is also worth noting that the proportions of the diagnosed sub-stages did not change across the eras, with almost half of the cases presenting with stage IIIA during each time-period. This confirms that the improvements in survival seen in our results were not a result of possible stage migration. Our sensitivity analysis also excluded the potential bias arising from the different classifications of the sub-stages during our study period.

The key strength of our study is the broad, representative sample size, spanning six German population-based registries over 12 years, representing a population of around 22.4 million (~ 27% of the entire German population). This is particularly valuable since clinical trials often focus on younger, healthier patients, potentially introducing selection bias. To address the high proportions of death certificate only (DCO) cases within the state registries, we excluded registries with DCO proportions exceeding the recommended 13% to ensure reliable survival estimates. We also addressed potential bias arising from different registry documentation methods. However, our study faces limitations due to lack of data on tumor operability and patient profile, which are crucial in deciding the treatment approach, including the option of surgery. Consequently, we could not ascertain whether the absence of surgery was due to inoperability or patient’s unfitness. Furthermore, our dataset did not include information on radiation dosage, surgical procedure details, chemotherapy type, treatment intent (curative/palliative), or treatment delivery date. To circumnavigate these limitations, we only included treated cases in our analysis and we grouped the cases diagnosed within a 3-years time period according to the treatment combination received.

Despite these limitations, our study provides valuable insights into the evolution of stage III NSCLC treatment patterns in Germany over a 12-years span. This knowledge is critical as it helps in understanding how changes in treatment modalities, diagnostic tools, and other factors may impact overall survival outcomes. As treatments continue to progress, it will be crucial to conduct further research to fully understand the interplay of these variables and how they can be optimized to improve patient outcomes.

## Conclusion

Our results indicate an improvement in survival for stage III NSCLC patients in Germany. This improvement could be partly explained by the more extensive use of FDG-PET in tumor diagnosis, alongside a general improvement in treatment procedures. Considering how immunotherapy is currently incorporated in the treatment plan, further studies with access to extensive treatment information are recommended to measure improvements in survival across time.

### Supplementary Information

Below is the link to the electronic supplementary material.

Supplementary file1 (PDF 659 KB)

Supplementary file2 (PDF 516 KB)

Supplementary file3 (PDF 397 KB)

## Data Availability

This study was based on the German national cancer registry data. The authors do not own these data and hence are not permitted to share them in the original form (only in aggregate form, eg, publications). Data were provided by the Robert Koch Institute (RKI).
