# Supplementary material for: Treatment patterns in stage III non‑small‑cell lung cancer patients: a population‑based study using German cancer registry data
Source: J Cancer Res Clin Oncol. 2023 Aug 30;149(17):15489–97. doi: 10.1007/s00432-023-05289-7 (PMC10620268; doi:10.1007/s00432-023-05289-7)
Supplement: Supplementary file 1 — Supplementary file1 (PDF 659 KB) [file 432_2023_5289_MOESM1_ESM.pdf]

Treatment Patterns in Stage III Non-small-cell lung cancer patients: a population-based study using German cancer registry data

Bedir, Ahmed[1]; Mehrotra, Sneha[2]; Gnüchtel, Jessica[3,4]; Vordermark, Dirk[1,3]; Medenwald, Daniel[1,3]

1. Department of Radiation Oncology, Health Services Research Group, University Hospital Halle (Saale), Ernst-Grube-Str. 40, 06120, Halle (Saale), Germany.
2. King's College London, Faculty of Life Sciences and Medicine, Guy's Campus, London SE1 1UL, United Kingdom.
3. Department of Radiation Oncology, University Hospital Halle (Saale), Ernst-Grube-Str. 40, 06120, Halle (Saale), Germany.
4. Department of Traumatology, Elisabeth-Hospital Leipzig, Biedermannstraße 84, 04277 Leipzig, Germany

Acknowledgments: None.

Address for correspondence:
Daniel Medenwald
Department of Radiation Oncology, University Hospital Halle (Saale),
Ernst-Grube-Str. 40, 06120, Halle (Saale), Germany.
Telephone no: +49-345-557-3453/4027
Email: Daniel.Medenwald@uk-halle.de

Appendix 1: Treatment percentages/Overall and median survival estimates

Table 1. Treatment received by NSCLC stage III patients 2007-2018.

	**Total**	**2007-2010**	**2011-2014**	**2015-2018**
**Number**	14606	3980	5493	5133
**Treatment received *n(%)***				
Surgery only	1843 (12.6)	488 (12.3)	617 (11.2)	738 (14.4)
Surgery + Radiotherapy	691 (4.7)	229 (5.8)	284 (5.2)	178 (3.5)
Surgery + Chemotherapy	1615 (11.1)	428 (10.8)	608 (11.1)	579 (11.3)
Radiotherapy only	2849 (19.5)	881 (22.1)	1050 (19.1)	918 (17.9)
Radiotherapy + Chemotherapy	3853 (26.4)	984 (24.7)	1582 (28.8)	1287 (25.1)
Chemotherapy only	2247 (15.4)	615 (15.5)	752 (13.7)	880 (17.1)
Surgery + Radiotherapy + Chemotherapy	1508 (10.3)	355 (8.9)	600 (10.9)	553 (10.8)

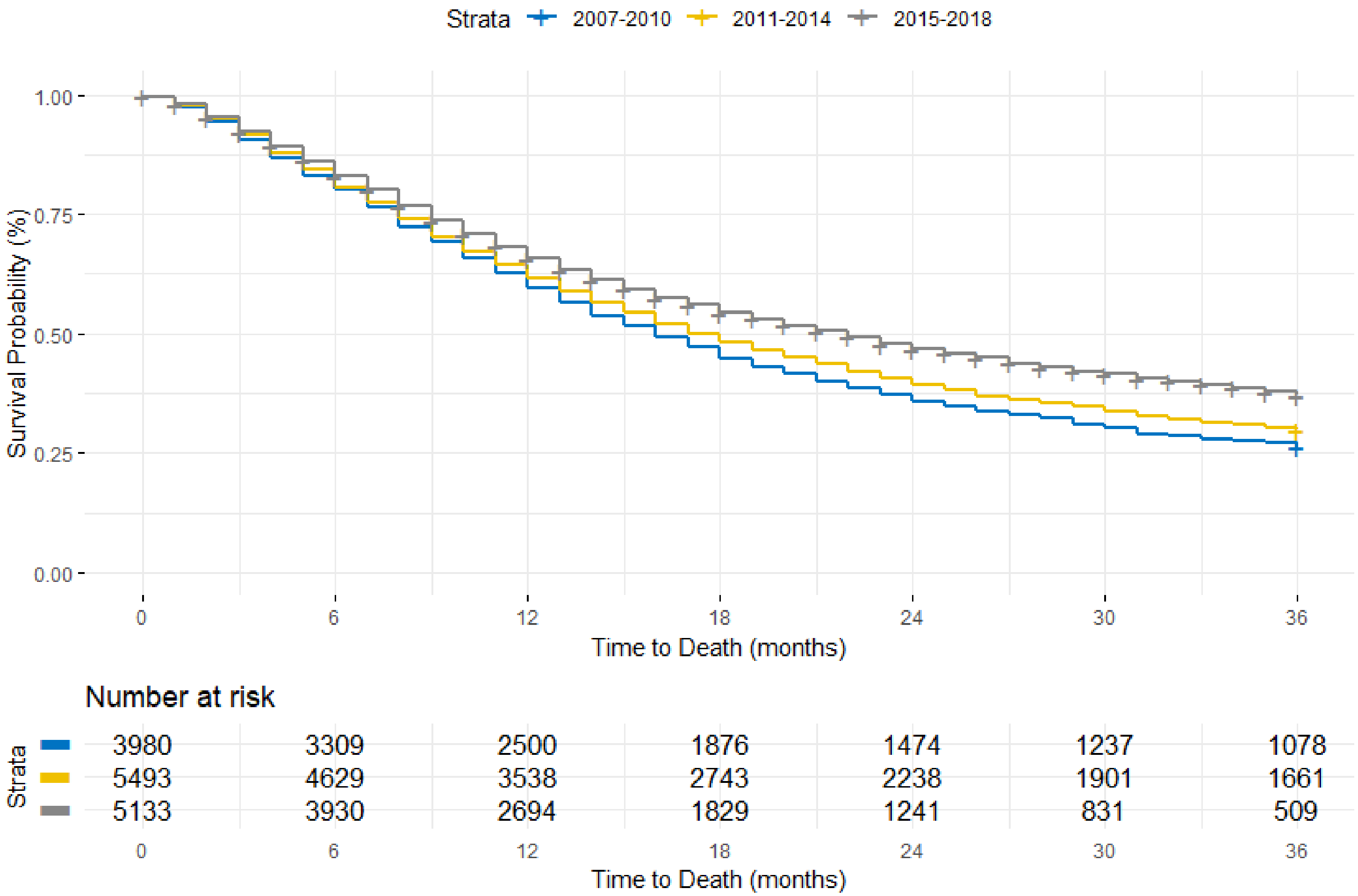

Figure 1: Kaplan Meier Curves comparing 3-year survival according to time-periods.

Table 2. 3-year survival rates of NSCLC stage III patients 2007-2018 according to treatment received.

	**2007-2010**	**2011-2014**	**2015-2018**
**Number**	3980	5493	5133
**Treatment received**			
Surgery only	37.9 (33.8-42.5)	38.1 (34.4-42.2)	47.3 (42.7-52.5)
Surgery + Radiotherapy	26.6 (21.5-33.0)	41.2 (35.9-47.3)	40.1 (31.6-50.8)
Surgery + Chemotherapy	51.6 (47.1-56.6)	53.5 (49.6-57.6)	58.7 (53.5-64.5)
Radiotherapy only	12.3 (10.3-14.6)	14.3 (12.3-16.6)	18.9 (15.7-22.8)
Radiotherapy + Chemotherapy	21.9 (19.5-24.7)	26.5 (24.4-28.8)	34.5 (31.1-38.3)
Chemotherapy only	14.6 (12.1-17.7)	15.2 (12.8-18.0)	25.2 (21.5-29.7)
Surgery + Radiotherapy + Chemotherapy	45.6 (40.7-51.1)	46.0 (42.2-50.2)	53.8 (48.5-59.7)

Table 3. Median survival estimates of NSCLC stage III patients 2007-2018 according to sub-stage.

	**Total**	**2007-2010**	**2011-2014**	**2015-2018**
**Number**	14606	3980	5493	5133
**IIIA**				
Number	6524	1779	2452	2293
Median OS *months (95%CI)*	25 (24-26)	22 (20-23)	24 (23-26)	32 (29-36)
3-year survival *% (95%CI)*	39.8 (38.6-41.2)	34.9 (32.7-37.1)	39.6 (37.7-41.5)	46.7 (44.1-41.5)
**IIIB**				
Number	5696	1524	2132	2040
Median OS	16 (15-16)	14 (14-16)	15 (14-16)	18 (17-20)
3-year survival	25.1 (23.9-26.3)	21.1 (19.1-23.3)	24.3 (22.6-26.2)	30.5 (27.9-33.5)
**IIIC**				
Number	2386	677	909	800
Median OS	12 (12-13)	11 (10-12)	12 (11-13)	13 (12-15)
3-year survival	17.8 (16.1-19.5)	15.1 (12.6-18.0)	16.2 (13.9-18.8)	25.6 (21.7-30.1)
