# Supplementary material for: Treatment patterns in stage III non‑small‑cell lung cancer patients: a population‑based study using German cancer registry data
Source: J Cancer Res Clin Oncol. 2023 Aug 30;149(17):15489–97. doi: 10.1007/s00432-023-05289-7 (PMC10620268; doi:10.1007/s00432-023-05289-7)
Supplement: Supplementary file 3 — Supplementary file3 (PDF 397 KB) [file 432_2023_5289_MOESM3_ESM.pdf]

Treatment Patterns in Stage III Non-small-cell lung cancer patients: a population-based study using German cancer registry data

Bedir, Ahmed[1]; Mehrotra, Sneha[2]; Gnüchtel, Jessica[3,4]; Vordermark, Dirk[1,3]; Medenwald, Daniel[1,3]

1. Department of Radiation Oncology, Health Services Research Group, University Hospital Halle (Saale), Ernst-Grube-Str. 40, 06120, Halle (Saale), Germany.
2. King's College London, Faculty of Life Sciences and Medicine, Guy's Campus, London SE1 1UL, United Kingdom.
3. Department of Radiation Oncology, University Hospital Halle (Saale), Ernst-Grube-Str. 40, 06120, Halle (Saale), Germany.
4. Department of Traumatology, Elisabeth-Hospital Leipzig, Biedermannstraße 84, 04277 Leipzig, Germany

Acknowledgments: None.

Address for correspondence:
Daniel Medenwald
Department of Radiation Oncology, University Hospital Halle (Saale),
Ernst-Grube-Str. 40, 06120, Halle (Saale), Germany.
Telephone no: +49-345-557-3453/4027
Email: Daniel.Medenwald@uk-halle.de

Appendix 2: Sensitivity analysis

Table 1: Hazard ratios comparing patients diagnosed in 2011-2014 and 2015-2018 to reference group (2007-2010) stratified according to treatment received. Cox models adjusted for age, sex, histology, and cancer sub-stage while adjusting for cancer registry

	**Surgery only**	**Surgery + Radiotherapy**	**Surgery + Chemotherapy**	**Radiotherapy only**	**Radiotherapy + Chemotherapy**	**Chemotherapy only**	**Surgery + Radiotherapy + Chemotherapy**	**Total***
**2007-2010**								
**2011-2014**	1.07 (0.91-1.25)	0.67 (0.53-0.83)	0.96 (0.80-1.15)	0.95 (0.86-1.04)	0.91 (0.83-1.01)	1.05 (0.93-1.18)	0.95 (0.89-1.15)	0.95 (0.91-1.00)
**2015-2018**	0.79 (0.67-0.92)	0.77 (0.58-1.01)	0.86 (0.69-1.07)	0.85 (0.76-0.94)	0.75 (0.66-0.84)	0.81 (0.72-0.92)	0.80 (0.65-1.00)	0.80 (0.75-0.85)

* Adjusted for treatment in addition to baseline variables

Table 2: Sub-stage (7th edition definition) stratified hazard ratios comparing patients diagnosed in 2011-2014 and 2015-2018 to reference group (2007-2010) stratified according to treatment received. Cox models were adjusted for age, sex, and histology.

	Surgery only	Surgery + Radiotherapy	Surgery + Chemotherapy	Radiotherapy only	Radiotherapy + Chemotherapy	Chemotherapy only	Surgery + Radiotherapy + Chemotherapy	Total*
**IIIA**								
**2007-2010**								
**2011-2014**	1.03 (0.88-1.21)	0.61 (0.48-0.79)	1.00 (0.81-1.22)	0.94 (0.82-1.08)	0.84 (0.72-0.99)	1.13 (0.93-1.37)	0.98 (0.78-1.22)	0.914(0.87-1.00)
**2015-2018**	0.76 (0.64-0.91)	0.70 (0.51-0.97)	0.80 (0.62-1.03)	0.86 (0.73-1.00)	0.72 (0.60-0.87)	0.80 (0.65-0.98)	0.81 (0.62-1.05)	0.78 (0.71-0.84)
**IIIB**								
**2007-2010**								
**2011-2014**	1.42 (0.94-2.15)	0.89 (0.56-1.41)	0.85 (0.56-1.24)	0.94 (0.82-1.08)	0.94 (0.84-1.06)	0.96 (0.83-1.11)	1.01 (0.81-1.50)	0.96 (0.89-1.02)
**2015-2018**	0.94 (0.61-1.45)	0.96 (0.56-1.64)	0.99 (0.67-1.47)	0.83 (0.72-0.97)	0.74 (0.64-0.84)	0.75 (0.64-0.88)	0.94 (0.66-1.33)	0.79 (0.72-0.86)

* Adjusted for treatment in addition to baseline variables
